# The decoupling between hemodynamic parameters and neural activity implies a complex origin of spontaneous brain oscillations

**DOI:** 10.3389/fncom.2023.1214793

**Published:** 2023-07-31

**Authors:** Ming Li, Lihua He, Zhuo Zhang, Zhen Li, Xuan Zhu, Chong Jiao, Dewen Hu

**Affiliations:** College of Intelligence Science and Technology, National University of Defense Technology, Changsha, China

**Keywords:** spontaneous oscillations, local field potential, optical imaging, nitric oxide synthase inhibitor, signal decoupling

## Abstract

**Introduction:**

Spontaneous low-frequency oscillations play a key role in brain activity. However, the underlying mechanism and origin of low-frequency oscillations remain under debate.

**Methods:**

Optical imaging and an electrophysiological recording system were combined to investigate spontaneous oscillations in the hemodynamic parameters and neuronal activity of awake and anesthetized mice after N^ω^-nitro-L-arginine methyl ester (L-NAME) administration.

**Results:**

The spectrum of local field potential (LFP) signals was significantly changed by L-NAME, which was further corroborated by the increase in energy and spatial synchronization. The important finding was that L-NAME triggered regular oscillations in both LFP signals and hemodynamic signals. Notably, the frequency peak of hemodynamic signals can be different from that of LFP oscillations in awake mice.

**Discussion:**

A model of the neurovascular system was proposed to interpret this mismatch of peak frequencies, supporting the view that spontaneous low-frequency oscillations arise from multiple sources.

## 1. Introduction

Brain oscillations are energetically the most efficient way of synchronizing neurons and forming neuronal assemblies (Buzsáki, 2023). These rhythmic oscillations are believed to be the carrier of communication between different brain areas (Palva et al., 2005; Schnitzler and Gross, 2005; Weiss et al., 2023). In particular, the ultraslow spontaneous oscillations of hemodynamic signal correlation are thought to be the manifestation of functional connectivity between distant regions (Biswal et al., 1995; He et al., 2004). Some temporally synchronized low-frequency fluctuations send messages across the corpus callosum and possibly carry messages synchronizing activity between symmetric bilateral functional regions (Biswal et al., 1995; Biswal and Hudetz, 1996). Moreover, spontaneous oscillations have shown more clinical and pathological correlations than previously thought. Numerous studies have demonstrated that abnormal spontaneous oscillations appear in brains with neuronal damage, cognitive impairments or neurological diseases (Pires and Earley, 2018; Guan et al., 2019; Koelewijn et al., 2019; Giustiniani et al., 2023). In addition, recent research has highlighted the effect of spontaneous low-frequency oscillations on neurological disorders. A coherent pattern of oscillating electrophysiological, hemodynamic, and cerebrospinal fluid dynamics during sleep has been discovered and is related to the clearance of metabolic waste products from the brain (Fultz et al., 2019; van Veluw et al., 2020).

The origin of spontaneous low-frequency oscillations remains an open issue (Drew et al., 2020). In early studies, hemodynamic oscillation signals were conceptualized as vascular oscillations that were generally considered to be myogenic in origin and independent of neural or other physiological activity (hemodynamic source) (Hundley et al., 1988). Using optical imaging (OI), Mayhew et al. (1996) reported a “0.1 Hz oscillation,” which is present across physiological states and species. More recently, it has been widely recognized that spontaneous hemodynamic signals are correlated with neural activity (suggesting that they originate from a neural source) (Julien, 2006). Significant low-frequency oscillation was observed in the power fluctuation of the local field potential (LFP) and exhibited a strong correlation between electrode pairs (Leopold et al., 2003). In addition, the extensive innervation of cerebral blood vessels indicates that neural activity may directly control local vasodilation/constriction (Krimer et al., 1998). The low-frequency oscillations of intracellular calcium and LFPs were similar to vascular signals in the rat somatosensory cortex, indicating that there is a common signal between blood oscillations and neuronal activity (Du et al., 2014). Furthermore, spontaneous infraslow activity (<0.1 Hz) in the brain was synchronized among OI, calcium imaging, and electrophysiology, suggesting that infraslow activity is the reflection of neurophysiological processes in the hemodynamic parameters (Mitra et al., 2018). The Granger causality relationships from LFP signals to cerebral blood flow also suggest that the spontaneous cerebral blood flow oscillations come from neural activity (Huang et al., 2014).

Accordingly, neural activity oscillations give rise to hemodynamic oscillations through neurovascular coupling, which describes the hemodynamic parameter that responds to local brain region activity to meet neuronal metabolism (Furchgott and Zawadzki, 1980; Chen et al., 2014; Dunn and Nelson, 2014). Thus, inhibiting neurovascular coupling is an intuitive way to explore the origin of spontaneous oscillations. This was accomplished using N^ω^ -nitro-L-arginine methyl ester (L-NAME) or 7-nitroindazole (7-NI) to block nitric oxide (NO) (Dirnagl et al., 1993; Lindauer et al., 1999; Matsuura and Kanno, 2002). Dirnagl et al. (1993) discovered that the low-frequency oscillations of cerebral blood flow were enhanced in anesthetized rats after interference with this coupling between the hemodynamic parameters and neural activity. Regular period oscillations appeared due to the inhibition of NO synthase (NOS), and the oscillations were markedly reduced after NO donor administration (Lindauer et al., 1999). However, in electrophysiological studies, the inhibition of NO synthase did not result in any changes in LFP signals (Matsuura and Kanno, 2002), which is not consistent with the hypothesis of neural origins.

Due to the lack of simultaneous recordings of hemodynamic parameters and neural activity, it remains unclear how hemodynamic oscillations are coupled with neural oscillations. In addition, anesthesia strongly disrupts neurovascular coupling (Goense and Logothetis, 2008; Pisauro et al., 2013; Gao et al., 2017) and the functional connectivity of the brain (Wu et al., 2017). Therefore, we made simultaneous recordings of the spontaneous oscillations of hemodynamic and neural activity in awake mice and compared the oscillations in the awake state with those in the anesthetized state with systemic administration of L-NAME. The primary finding was the decoupling between the hemodynamic parameters and neural activity, which indicates the complex origin of spontaneous brain oscillations.

## 2. Materials and methods

This study was performed in strict accordance with the recommendations of the Guide for the Care and Use of Laboratory Animals of the National Institutes of Health and was approved by the Medical Ethics Committee of 921 Hospital.

### 2.1. Animal preparation and experimental model

**FIGURE 1:**
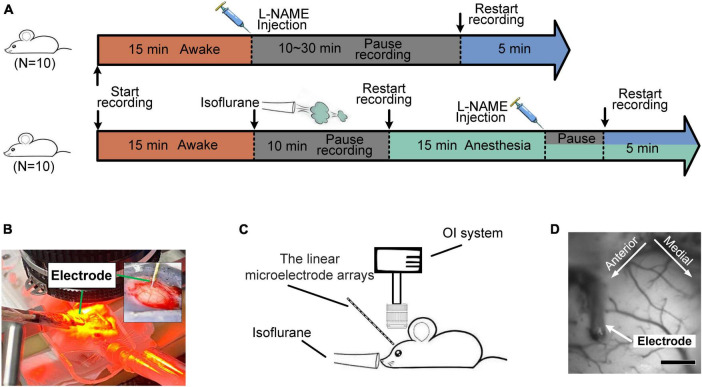
Diagrams of the experiments for the hemodynamic and electrophysiological signals. **(A)** Flow chart of the experiment on awake (top) and anesthetized (bottom) mice. Orange segment: awake; blue: after the injection of L-NAME; green: anesthesia with isoflurane; gray: a pause in recording for a rest period. **(B,C)** Diagram of the experimental setup. The inset graph in panel **(B)** is a magnified view of the electrode implant location. **(D)** Light reflection image recorded from the somatosensory cortex. Scale bar: 500 μm.

Before the experiment, 35 male adult Kunming mice (40 ± 3 g) were fed a standard diet and water in a room kept at 22 ± 2°C on a 12/12-h light/dark cycle. The mice were randomly divided into two groups corresponding to the awake (*n* = 25) and anesthetized states (*n* = 10). For mice in the awake state, spontaneous oscillation signals were recorded before and after the injection of L-NAME (I.P., 50 mg/kg) (Figure 1A top). For mice in the anesthetized state, we also recorded the signals before/after the injection of L-NAME (Figure 1A bottom).

In surgery, a thinned skull window was made over the somatosensory cortex with a modified method for producing reinforced thinned skull windows (Drew et al., 2010). Briefly, mice were anesthetized with isoflurane (1.5% in oxygen) and fixed in the stereotaxic apparatus, with their eyes covered in ointment. The skulls were exposed and cleaned with saline. A large area over the right somatosensory cortex was coarsely thinned using a high-speed handheld drill. Then, a fine thinning procedure was performed to remove the ridges and smooth the skull. During thinning, saline was constantly applied to prevent heating. Afterward, the skull was briefly vacuum dried to remove the saline. A drop of transparent superglue was applied to the thinned region, and a glass coverslip was attached. Through the covers, the cortical surface and pial vessels were clearly visible. Finally, dental cement was used to glue a customized metal head bar to the skull and cover the exposed skull.

Before data collection, mice recovered from surgery for at least 5 days, followed by a training period of 3–5 days during which they were acclimated to head fixation and sitting in the tube. For electrophysiological recordings, covered glass, thinned bone and dura were removed under isoflurane anesthesia (1.5% in oxygen). Then, a linear microelectrode array was implanted, and agarose (1%) with a low melting point was applied to cover the exposed cortex. For synchronized recording of optical and electrophysiological signals (Chernov et al., 2016), a cover glass was carefully attached over the agarose. After the operation, anesthesia was retreated, and mice recovered for at least 20 min before data acquisition. For data acquisition in the anesthetized state, isoflurane (1.2% in oxygen) was continuously administered, and the body temperature of the mice was maintained at 37.5°C. Details on the process of surgery and data observation are described in previous studies (Li et al., 2007; Huang et al., 2014).

### 2.2. Optical imaging and electrophysiological recording

The OI technique was used to acquire hemodynamic signals, while electrophysiological recording was used to acquire neural activity (Figures 1B, C). The OI signals (Pouratian et al., 2003) were recorded by the Optical Imaging system (Imager 3001; Optical Imaging Inc., Germantown, New York, USA). The OI system consists of three main parts: a high-precision charge-coupled device (CCD) (12-bit, 1,024 × 1,024 pixels, 60 dB), a cold light source and an optical path (Figure 1D). In this work, red illumination (605 ± 10 nm), which is sensitive to changes in deoxyhemoglobin (Hb), was used to capture the primary somatosensory cortex signals.

For electrophysiology recordings (Sharott, 2013), a linear microelectrode array (1.5 mm 16-channel, Gaithersburg, MD, USA) that was connected to a Cerebus data acquisition system (Cyberkinetics Neurotechnology Systems, Inc.) was implanted at an angle of 30°∼45° into the cortex under direct visualization with a surgical stereoscope. The LFP signals were obtained using an amplifier with a high-pass filter (Intan RDH2132). All recordings were made in a completely dark room.

### 2.3. Data analysis

#### 2.3.1. Preprocessing

For the OI data analysis, the mean time series was obtained by averaging the gray level of the pixels within the region of interest (ROI). The ROI was usually selected from the cortex near the electrode and without large vessels. For the LFP data analysis, data from the electrode channel located at the lens focus of the optical instruments were selected. Because the depth of focus of our OI system is 500 μm, only the data from the electrode channel with a depth of ∼500 μm were selected for further analysis. For the correlation analysis between LFP and OI signals, the LFP was preliminarily downsampled to the same sample rate as that of OI by linear interpolation. For the comparison of spectra between LFP and OI, the noise was removed by taking the moving of the signals with a window of 1 s, and the drift fluctuation, which was calculated by smoothing the time series with a window of 10 s, was removed. For the comparison of waveforms, the signals were both normalized to a zero mean and unit standard deviation.

#### 2.3.2. Analysis of the amplitude-frequency spectrum

The traditional fast Fourier transform method (Cooley and Tukey, 1965) was used to obtain the amplitude-frequency spectrum (in the range <0.5 Hz, defined as the low-frequency band) of the OI and electrophysiological signals. The energy of the LFP and OI signals was used to evaluate the oscillation strength. The energy in the band [α β] is defined as


(1) $$E_{\alpha \,\beta }=\sum_{k_{\alpha }}^{k_{\beta }}X\,\left(k\right)\cdot X^{*}\,\left(k\right).$$

Where *X* is the discrete Fourier transform of the signal; the asterisk denotes complex conjugation; and*k*_α_ = α*N*/*f*_*s*_, *k*_β_ = β*N*/*f*_*s*_, where *f*_*s*_ and *N* are the sample frequency and the sample number of the signal, respectively.

To identify the presence or absence of peaks in the frequency spectrum, the crest factor (CF), which is defined as the logarithmic ratio of the maximum value to the root mean square of the frequency spectrum, was calculated. The crest factor is defined as


(2) $$C=\log\left(\max\left(X\,\left(k\right)\right)/\sqrt{E_{\alpha \,\beta }/N}\right).$$

Furthermore, the exact peak frequency was estimated using the multitaper analysis method (MTM) of spectral analysis, which generated the MTM power spectrum and recorded all the highlighted frequency values (Thomson, 2007). It provides a novel means of spectral estimation (Thomson, 1982; Percival and Walden, 1996) of a time series that is believed to exhibit a spectrum containing both continuous and singular components. To reject false oscillations, i.e., harmonics that have a large amplitude but do not correspond to a periodic signal, the F test was used to test the confidence of identified frequencies (Lomax, 2007). Of the identified frequencies, only those whose F-values exceeded the threshold corresponding to a 95% confidence level were considered valid peak frequency candidates.

#### 2.3.3. Analysis of synchronization between LFP and OI

To evaluate the synchronization level between LFP and hemodynamic signals, the correlations between the two signals were calculated over a duration of 30 s. Because the sampling frequency is different from that of OI, LFP signals were preliminarily downsampled to the sampling rate of OI. Since the hemodynamic signal lags behind the electrophysiological signal (Yu et al., 2012), the hemodynamic signals were moved ahead by 2.5 s before correlation calculation (de Zwart et al., 2005). In this work, the absolute value of the Pearson correlation coefficient was used (Lee Rodgers and Nicewander, 1988). Because synchronized signals are strongly correlated, correlation analysis can provide a direct evaluation of the synchronization between LFP and OI signals.

To further study the synchronization between LFP and OI signals, short-time Fourier transform (STFT) analysis (Kehtarnavaz, 2008) was also carried out. STFT is a time-frequency analysis algorithm that can illustrate the time-localized frequency information for situations in which the frequency components of a signal vary over time. Because two desynchronized signals have different time-frequency spectra, by comparing the frequency fluctuations of LFP and OI signals at different times, we can intuitively evaluate the synchronization level between the two signals. In this work, a Hanning-tapered window with a length of 25.6 s (256 sampling points) was used to divide the signal into segments and perform windowing.

All the data analysis in this work was carried out using MATLAB (MathWorks, Natick, MA, USA). The figures were generated with MATLAB, and the insertion and adjustment of labels and some text in figures were performed in VISIO. Statistical significance was determined with a two-sample *t*-test (Box, 1987).

## 3. Results

In this study, the hemodynamic parameters and neural activity were recorded separately by OI and electrophysiological recording. The effects of L-NAME on spontaneous oscillations in the two experimental models (awake and anesthetized) were investigated.

### 3.1. L-NAME administration led to a peak frequency in awake mice

**FIGURE 2:**
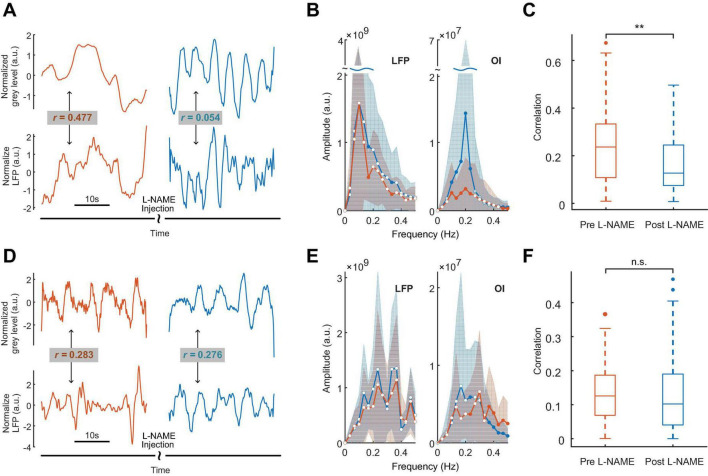
The effects of L-NAME on oscillations of both hemodynamic and LFP signals. Panels **(A–C)** for awake mice, **(D–F)** for anesthetized mice. Orange and blue represent before and after the injection of L-NAME, respectively. **(A)** An example of hemodynamic (upper panel) and LFP (lower panel) signals before and after the injection of L-NAME. Low-frequency oscillations were induced after L-NAME administration. Following the injection of L-NAME, the correlation between hemodynamic and LFP signals decreased remarkably (R-value: 0.477 vs. 0.054). **(B)** Group statistics of the power spectrum of LFP (left) and OI (right) signals for awake mice. The solid curves are the mean power spectrum, and the shaded areas represent the standard deviation. The filled-circle markers indicate significant differences before and after L-NAME (*p* < 0.05), and the hollow circles indicate no significance. **(C)** A statistical comparison of the correlation between LFP and hemodynamics. L-NAME administration induces a significant decrease in the correlation. The median value drops from 0.24 to 0.13. **Indicates significance at the 0.01 level. **(D)** An example from anesthetized mice. The correlations of hemodynamic (upper panel) and LFP (lower panel) signals are 0.283 and 0.276, respectively. **(E)** Group statistics of the power spectra of LFP and OI signals for anesthetized mice. The solid curves are the mean power spectra, and the shaded areas represent the standard deviation. The filled-circle markers indicate significant differences before and after L-NAME (*p* < 0.05), and the hollow circles indicate no significance. **(F)** A statistical comparison of correlation. In anesthetized mice, L-NAME administration did not change the correlation significantly. The correlations are relatively small, and the median values are 0.13 and 0.10, respectively. n.s. indicates no significant difference.

**FIGURE 3:**
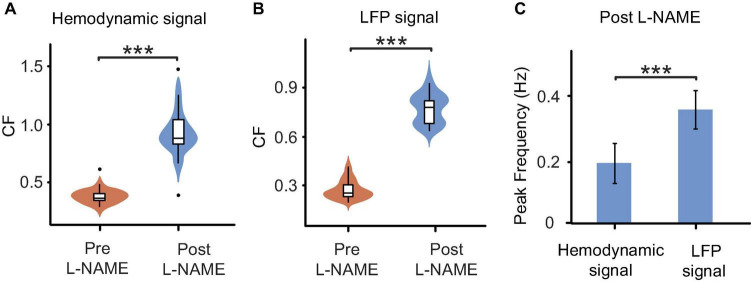
Comparisons of oscillations before and after L-NAME administration in all awake mice. **(A)** Boxplot of the CF coefficients of hemodynamic signals. The CF coefficients after the injection of L-NAME (blue) are significantly larger than those before injection (orange), which illustrates that oscillations were significantly enhanced by L-NAME (*P* < 0.001). **(B)** Boxplot of the CF coefficients of LFP signals. The CF coefficients showed similar increases after the injection of L-NAME (*P* < 0.001). The shadow beside the boxplot indicates the value distribution. **(C)** Comparisons of the peak frequency between hemodynamic and LFP oscillations after the injection of L-NAME. L-NAME triggered oscillations in both hemodynamic parameters and neural activity; however, the two oscillations occurred at different frequencies (*P* < 0.001). This result implies decoupling hemodynamic and LFP oscillations. Violin plots behind the boxplots represent the distributions of CF values. ***Indicates significance at the 0.001 level.

The analysis of spontaneous oscillations in this study revealed that the hemodynamic parameters and neural activity of mice could be affected by L-NAME administration, which were addressed separately in awake mice (Figure 1A top). After L-NAME administration, blood vessel contraction was clearly observed by the OI system during the experiment, indicating that NO synthase was inhibited. The time series of hemodynamic signals exhibited regular periodic fluctuation (Figure 2A, upper panel). The amplitude was enhanced significantly at most low-frequency points among all subjects (Figure 2B, right; the filled-circle markers indicate significant differences, *p* < 0.05). The time series of periodic LFP also showed enhanced fluctuation after the injection of L-NAME (Figure 2A, lower panel). The statistical power spectra before and after L-NAME are shown in Figure 2B (left). At most frequencies, the increase in power was not significant. The correlation of hemodynamic and LFP signals decreased remarkably following the injection of L-NAME, and the R-value dropped from 0.477 to 0.054. Figure 2C shows the statistical comparison of the correlation before and after L-NAME administration. L-NAME induced a significant decrease in the correlation (*p* < 0.01), and the median value dropped from 0.24 to 0.13. The CF analysis suggested that both hemodynamic and LFP signals had the same trend of significant increase following the injection of L-NAME (Figures 3A, B). Therefore, after NO synthase was suppressed, the relationship between hemodynamic parameters and neural activity appeared to be interrupted, and spontaneous oscillations acquired different characteristics.

**FIGURE 4:**
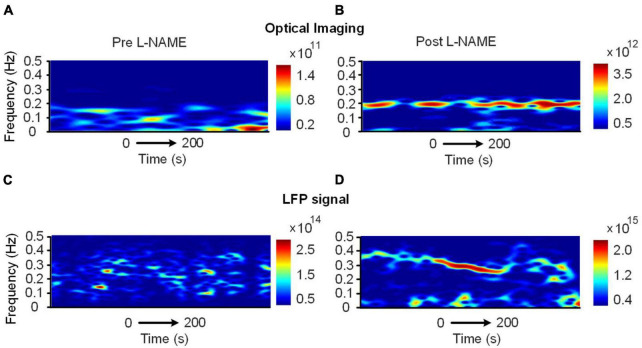
Time-varying spectrum of the low-frequency oscillations (<0.5 Hz) before and after the injection of L-NAME, as revealed by the STFT in awake mice. **(A,B)** The spectrum of hemodynamic signals, **(C,D)** the spectrum of the LFP signals synchronously recorded with panels **(A,B)**. Panels **(A,C)**: before L-NAME injection; Panels **(B,D)**: after injection. After the injection of L-NAME, a stable low-frequency oscillation was triggered, while the hemodynamic and LFP oscillations were not present in the same frequency band.

The correlation decrease in awake mice implies that hemodynamic and LFP signals may be decoupled by L-NAME administration. To further study this issue, STFT analysis was carried out for 200 s before and after the injection of L-NAME in awake mice. The spectrogram illustrated that the spontaneous LFP and hemodynamic oscillations peaked at different frequencies (Figure 3C). The peak frequency of hemodynamic signals was continuously centered at approximately 0.2 Hz (Figures 4A, B), while LFP signal peaks appeared at different frequencies (centered at 0.3–0.4 Hz) over time after the injection of L-NAME (Figures 4C, D). This showed decoupling of hemodynamic and LFP oscillations.

### 3.2. L-NAME enhanced LFP amplitude in different frequency bands and synchronization across space in awake mice

To investigate the effect of L-NAME on the electrophysiological signals in higher frequency bands, we calculated the energy of LFPs in 5 frequency bands: the delta band (1–4 Hz), the theta band (4–8 Hz), the alpha band (8–12 Hz), the beta band (12–40 Hz) and the gamma band (40–80 Hz). Approximately 20% of awake mice showed significant increases in energy in all frequency bands, and the increases were largest in the delta band. The results showed that L-NAME has non-negligible effects on a wide range of neural activity and does not exclusively affect low-frequency oscillations.

**FIGURE 5:**
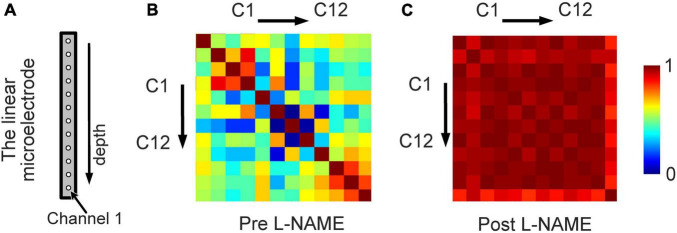
An increase in the synchronization of the LFP from different locations owing to L-NAME administration. **(A)** Schematic illustration of the 12-channel distribution from different positions of the linear microelectrode arrays. **(B)** The correlation coefficient matrix of the LFP signals from 12 channels for awake mice without L-NAME administration. **(C)** The correlation coefficient matrix after the injection of L-NAME. The correlation coefficients were larger after L-NAME administration, which means that the spontaneous oscillations of different locations became consistent.

To study the synchronized effect of L-NAME for different locations of the cortex, we calculated the similarity among the LFP signals recoded by 12 channels of the electrodes (Figure 5A). A matrix of Pearson correlation coefficients between all pairs of channels from deep to shallow is shown in Figures 5B, C. After L-NAME administration, the values in the correlation coefficient matrix were significantly increased. The features of spontaneous oscillations in LFP signals tend to be uniform in mice treated with L-NAME. In contrast, the features of the spontaneous oscillations are diverse and varied in awake mice among different locations of the somatosensory cortex. These results showed that the LFP signals were previously disordered and became concordant after the injection of L-NAME.

### 3.3. Spontaneous oscillations appeared to be unaffected by L-NAME in anesthetized mice

**FIGURE 6:**
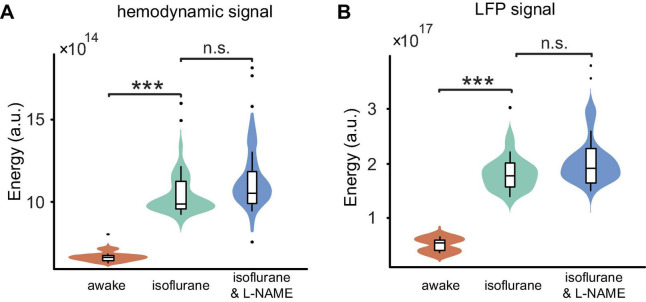
Comparisons of the energy among the three stages (awake, anesthesia, and anesthesia with injection of L-NAME). **(A)** Boxplot of the hemodynamic low-frequency oscillation energy. Under anesthesia (green), the energy was significantly larger than that of awake mice (orange) (*P* < 0.001), while the injection of L-NAME (blue) had no effect on the energy (*P* > 0.05) in anesthetized mice. **(B)** The energy of LFP low-frequency oscillation changes similar to hemodynamic signals throughout the three stages. Violin plots behind the boxplots represent the energy distributions. Error bars indicate the standard errors. ***Indicates significance at the 0.001 level. n.s., no significant difference.

As shown in Figures 2D–F, in anesthetized mice, L-NAME administration did not significantly change the power spectrum or the correlation between LFP and OI. Moreover, the correlations were relatively weak, with median values of 0.13 and 0.10, respectively. A weak correlation might mean that anesthetics also have the effect of decoupling LFP and hemodynamic signals, which is the same as L-NAME. To investigate the impact of anesthetics on the efficiency of L-NAME, the amplitude-frequency features of LFP and hemodynamic signals were analyzed. Previous studies have concluded that hemodynamic signals exhibit an increased peak frequency after L-NAME injection, while the amplitude-frequency features of LFP oscillations do not significantly change (Dirnagl et al., 1993; Matsuura and Kanno, 2002; Takuwa et al., 2012). Indeed, our previous studies demonstrated that the spontaneous low-frequency oscillation amplitudes of the hemodynamic signals increased with deeper levels of anesthesia, and we further speculate that neural activity also increased (Zhang et al., 2020). In our experiment, we performed simultaneous OI and electrophysiological recording in the somatosensory cortex of mice as they transitioned from the awake state to the anesthetized state (Figure 1A bottom). In the hemodynamic signals, the spontaneous oscillations became more regular after anesthesia (1.2% isoflurane), and the low-frequency oscillations were enhanced. As shown in Figure 6A, the energy of the low-frequency band appeared to be significantly increased (*P* < 0.001) after anesthesia. A similar impact of isoflurane anesthesia was also reflected in the LFP signals. The spontaneous oscillations of LFPs consistently changed after anesthesia, increasing in the low-frequency band, which was consistent with the changes observed in hemodynamic signals. The energy of the low-frequency band also significantly increased in anesthetized mice (*P* < 0.001, Figure 6B). The results implied that the impact of isoflurane on spontaneous low-frequency oscillations of hemodynamic and LFP signals was consistent. However, neural activity seems to be suppressed in anesthetized mice. The spike discharge of neurons was significantly reduced at the *p* < 0.001 level under anesthesia. Nevertheless, a significant coupling relationship between the spontaneous low-frequency oscillations of hemodynamic and LFP signals seems to exist.

In awake mice, we demonstrated that L-NAME has a significant effect on the hemodynamic and LFP signals. In anesthetized mice, we investigated the spontaneous oscillation features before and after the injection of L-NAME using an OI system and electrophysiology. Although an increase in amplitude at the peak frequency was found in hemodynamic signals after the injection of L-NAME, there were no significant differences (*P* > 0.05) in the data (Figure 6A). The amplitude-frequency spectrum of the LFP signals was not significantly changed after the injection of L-NAME, and the energy of the low-frequency band was basically similar (Figure 6B). Statistical results showed that L-NAME had no significant impact on the hemodynamic or electrophysiological signals in anesthetized mice.

### 3.4. Modeling of the neurovascular coupling system

The neurovascular coupling system must have two properties. First, it must increase the local blood supply quickly following local neural activation to supply sufficient oxygen and nutrients. Second, according to our experimental findings, the hemodynamic signals should show regular oscillations and decoupling from neural activation after NO blockage. Therefore, a neurovascular coupling model must possess the following characteristics. First, before NO blockage, the output (hemodynamic signals) can follow the input (neural activity) well. Second, after NO blockage, the output should be decoupled from the input and must show oscillations.

**FIGURE 7:**
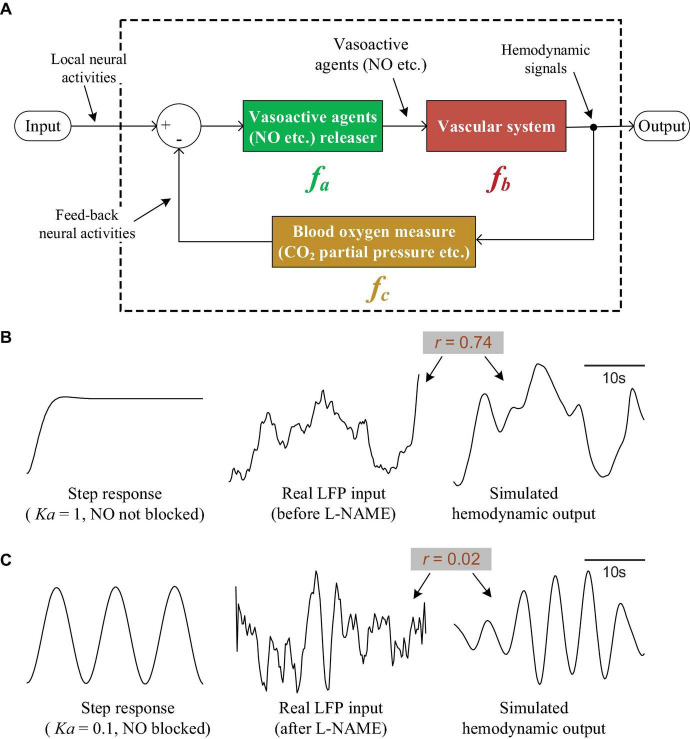
The neurovascular coupling model and simulations. **(A)** The theoretical neurovascular coupling model, which is designed as a control system with negative feedback. **(B)** The input/output characteristics of the model when NO is not blocked. The left curve is the step response of the system. The right curve is the response of the system when feeding a real LFP signal (the middle curve). **(C)** The input/output characteristics of the model when NO is blocked. Left: the step response. Right curve: the response of the system when feeding a real LFP signal (the middle curve).

To satisfy the above properties, we propose a neurovascular coupling model that is designed as a control system with negative feedback (Figure 7). In Figure 7A, module *f*_*a*_ represents the source of vasoactive agents, which is responsible for releasing vasoactive agents such as NO and then regulating vascular activity. Here, *f*_*a*_ is simply defined as a proportional component. Module *f*_*b*_ models the vascular system. Exposure to vasoactive agents will cause vasodilation/constriction and regulate hemodynamic parameters such as local blood flow and blood volume. According to previous works (Bumstead et al., 2017; Das et al., 2021; Lambers et al., 2023), vasomotion (an oscillation of vascular tone) is usually characterized as low-frequency oscillations and is not modulated by local neural activity, indicating that the vascular system may be an oscillating system in itself. Therefore, the vascular system is modeled as an oscillating component here. Module *f*_*c*_ is the sensor used to monitor the parameters related to local blood oxygen. It measures the local oxygen level and then feeds this information back to the input terminal. At the input terminal, the oxygen demand created by the local neural activity is integrated with the measured local blood oxygen; module *f*_*a*_ is then stimulated, followed by *f*_*b*_, to increase the blood oxygen until the local oxygen equals the requirements. With the feedback system, it is possible for the output to track the input accurately. If NO is blocked, the proportional component *f*_*a*_ will be nearly broken, and the vascular system *f*_*b*_ will almost be unaffected by the feedback and input; under these conditions, *f*_*b*_ will output oscillations. As an example to illustrate the feasibility of this model, the 3 modules are designed as


(3) $$f_{a}=K_{a},$$

(4) $$f_{b}=\frac{K_{b}}{s^{2}-\alpha \cdot s+\beta },$$

(5) $$f_{c}=K_{c}\cdot \frac{s+\gamma }{s+\varphi },$$

where *s* is the complex variable in the Laplace transform. Additionally, as an example, let *K*_*a*_ = 1, *K*_*b*_ = 2,*K*_*c*_ = 10,α = 0.2,β = 1.5,φ = 10,γ = 0.1, and plot the step response of the whole model (the left curve in Figure 7B). It can be seen that the response is able to track the step signal well, indicating that the system output can follow the input accurately. To further verify the utility of our model for interpreting the experimental results, we fed a real LFP signal (the middle curve in Figure 7B, also shown in Figure 2A, the lower orange curve) into the system and compared the output (the right curve in Figure 7B) with the real hemodynamic signal. The simulated output is quite similar to the real hemodynamic signal (the upper orange curve in Figure 2A). The correlation coefficient between the input LFP and output hemodynamic signal is 0.74, suggesting that the simulated hemodynamic signal tracks the input well when NO is not blocked.

To simulate NO blockage, we reduced *K*_*a*_ to 0.1; the step response became an oscillation (the left curve in Figure 7C), indicating that the output was decoupled from the step input and showed oscillations. By taking a real LFP signal obtained under NO blockage (the middle curve in Figure 7C, also shown in Figure 2A, the lower blue curve) and feeding it into the system, we obtain the simulated hemodynamic signal (the right curve in Figure 7C), which shows obvious oscillations. The correlation between the LFP and hemodynamics drops to 0.02, suggesting that the hemodynamics decouple from the LFP after NO blockage.

## 4. Discussion

Previous studies have addressed the importance of spontaneous oscillations in hemodynamic and neural signals. An inherent relationship between hemodynamic signals and neural activity has been reported (Huang et al., 2014; Chow et al., 2020). One of the objectives of this study was to further compare spontaneous oscillations between these two signals after the injection of L-NAME. In our research, the role of NO in spontaneous low-frequency oscillations was investigated in awake mice, and the coupling between hemodynamic parameters and neural activity was focused on. Moreover, we carried out an in-depth study on the effect of L-NAME on spontaneous signals in anesthetized mice. The major findings include the following: First, systemic administration of L-NAME in awake mice was found to cause regular hemodynamic and LFP fluctuations, but the peak frequency was different between the hemodynamic and LFP signals. In addition, isoflurane increased the low-frequency oscillation energy in hemodynamic signals. This result is consistent with recent research showing the considerable impact of isoflurane on hemodynamic signals (Zhang et al., 2020). Interestingly, the same enhancement occurs in the spontaneous low-frequency oscillations of LFP signals. However, after the injection of L-NAME in anesthetized mice, no significant effect on the spontaneous oscillations could be observed.

### 4.1. The effect of L-NAME on spontaneous oscillations in awake mice

In anesthetized rats, an enhancement of low-frequency oscillations in cerebral blood flow has been observed after the injection of an NOS inhibitor (Ances et al., 2010). The low-frequency oscillations of hemodynamic signals retained a uniform oscillation frequency after the injection of L-NAME (Dirnagl et al., 1993; Matsuura and Kanno, 2002; Sardo et al., 2002). However, changes in LFP oscillations have not yet been found in anesthetized rats after the injection of L-NAME. In our results, a peak frequency in spontaneous LFP oscillations emerged among awake mice following the injection of L-NAME, which is similar to the oscillations in the hemodynamic signals. The enhanced spectrum energy and correlation further demonstrated the effect of L-NAME on the LFP spectrum. However, after the injection of L-NAME, the peak frequency of the low-frequency band was not obvious in partial channels (negative results of approximately 16%), and enhancements in energy and correlation were found in only 20% of experimental objects, which is likely one of the reasons that no significant results were found in previous studies. Although this significant effect is not easily observed, the effect of L-NAME on spontaneous neural activity is not negligible.

### 4.2. The effect of L-NAME on spontaneous oscillations in anesthetized mice

In our experiments, we used isoflurane to anesthetize mice to investigate spontaneous brain oscillations. Isoflurane is the most commonly used inhalation anesthetic in rodents and is known for a quick onset and offset of anesthesia. It is a potent vasodilator whose effects on vessels are opposite to the effects of L-NAME (Redfors et al., 2014). Some recent studies have reported that neural activity, vascular tone, and neurovascular coupling are altered by anesthesia and that hemodynamic spontaneous oscillations can be affected by the depth of anesthesia (Lalou et al., 2016; Keilholz et al., 2017; Zhang et al., 2020). However, the effect of anesthesia on spontaneous oscillations in LFPs remains unknown. In our current study, we found that isoflurane anesthesia enhanced the energy of the low-frequency band in the hemodynamic and LFP signals. Notably, the low-frequency band of these signals had a similar trend and peak frequency in anesthetized mice. These findings might contribute to our understanding of the relation between blood and neural activity. Previous studies have shown that the induced low-frequency oscillations of cerebral blood flow were enhanced after the injection of NOS inhibitors in anesthetized rats (Dirnagl et al., 1993; Matsuura and Kanno, 2002; Takuwa et al., 2012). In our studies, a similar procedure was used to examine spontaneous low-frequency oscillations in anesthetized mice via a simultaneous recording of hemodynamic signals and neural activity after the injection of L-NAME. In hemodynamic signals, although an enhanced amplitude was observed in some mice, no statistically significant enhancement of energy was found after the injection of L-NAME. This result is different from those of previous studies, as isoflurane is a vasorelaxant, L-NAME leads to vasoconstriction (Redfors et al., 2014), and the relaxant effect of isoflurane is far stronger than the constriction caused by L-NAME. Regarding LFP signals, significant changes were not obtained after L-NAME administration. Further investigations are needed to determine the effect of isoflurane on the efficiency of L-NAME.

### 4.3. The complex origin of spontaneous oscillations

The origin of spontaneous oscillations remains poorly understood. In early studies, the oscillations observed on the hemodynamic signals were considered to be myogenic in origin and independent of neural or other physiological activity (a hemodynamic source) (Hundley et al., 1988; Mayhew et al., 1996). More recently, it has been suggested that these hemodynamic oscillations originate from a neural source (Huang et al., 2014; Fultz et al., 2019; Chow et al., 2020). Normally, hemodynamic signals accurately follow neural activity; therefore, it is most likely that hemodynamic oscillations reflect underlying oscillating neural activity. In fact, abundant oscillations were found in the LFP signal, which is a common measure of neural activity (Merchant et al., 2015; Ray, 2015). Using a detailed computational model of the brain network, it was shown that spontaneous low-frequency oscillations of LFPs could originate from ion concentration dynamics (Krishnan et al., 2018). The ion concentration dynamics mediated by neuronal activity may contribute to the generation of spontaneous hemodynamic oscillations. However, neural activity may not be the only reason for vasomotor changes (Duyn, 2011; Longden et al., 2017). Studies (Bumstead et al., 2017; Das et al., 2021; Lambers et al., 2023) have shown that hemodynamic signals can emerge from vasomotion (an oscillation of vascular tone independent of heartbeat, respiration, and neuronal activity) and Mayer waves (global oscillations in arterial blood pressure correlated with sympathetic neural activity), i.e., the hemodynamic signal cannot be solely explained by neural dynamics, indicating that low-frequency oscillations can originate from the vascular system as well as neural activity.

In our experiment, we observed that L-NAME induced regular oscillations and that the peak oscillation frequency of LFPs and hemodynamic signals could be different. In other words, when we blocked NO and thus weakened the connection of vascular activity to neural activity, electrophysiological and hemodynamic signals oscillated independently. This implies that there are at least two oscillation sources, of which one is neural and one is hemodynamic (myogenic). It is most likely that both hemodynamic sources and neural sources exist, indicating the complex origin of spontaneous oscillations. The proposed neurovascular coupling model could interpret the relationship between the two sources: if module *f*_*a*_ works well, the hemodynamic signals follow the neural fluctuations, and then the neural source is observed; if *f*_*a*_ is weakened or blocked, the hemodynamic signals reflect the intrinsic oscillations of vascular tissue, allowing the myogenic source to be observed.

In summary, it seems that both the intrinsic oscillations of vascular tissue and neural activity contribute to the oscillations of hemodynamic signals. Our findings highlight that the relationship between neural activity and hemodynamic parameters is not straightforward; other processes may also modulate the hemodynamic signals. For example, astrocytes may be involved. As a key mediator of neurovascular coupling, astrocytes can release vasoactive agents to mediate vasodilation/constriction and regulate ultraslow arteriole oscillations based on the bidirectional communication between arterioles and astrocyte endfeet (Haidey et al., 2021). Intriguingly, the evoked and intrinsic astrocytic calcium signals are coupled to positive and negative BOLD signals, respectively. Moreover, the intrinsic astrocytic calcium signal is correlated with an increased power level of brain resting-state fluctuation on EEG (Wang et al., 2018). More efforts need to be made to elucidate the complicated mechanism of spontaneous low-frequency oscillations.

## Data availability statement

The raw data supporting the conclusions of this article will be made available by the authors, without undue reservation.

## Ethics statement

The animal study was reviewed and approved by the Medical Ethics Committee of 921 Hospital.

## Author contributions

LH, ML, and DH designed and supervised this study. LH, ZL, XZ, and CJ performed the animal surgeries and data acquisition. ML and ZZ performed the analysis. ML, LH, and ZZ wrote the manuscript. All authors read and approved the final manuscript.
